# A Novel Decision Aid to Encourage Smoking Cessation Among Patients at an Urban Safety Net Clinic

**DOI:** 10.5888/pcd15.180215

**Published:** 2018-10-11

**Authors:** Sumit D. Agarwal, Matthew Kerwin, Jacob Meindertsma, Andrew M.D. Wolf

**Affiliations:** 1 Division of General Internal Medicine and Primary Care, Department of Medicine, Brigham and Women’s Hospital; Harvard Medical School; Boston, Massachusetts; 2 Department of Medicine, University of Virginia School of Medicine, Charlottesville, Virginia; 3 Division of General, Geriatric, Palliative & Hospital Medicine, Department of Medicine University of Virginia School of Medicine, Charlottesville, Virginia

## Abstract

**Introduction:**

Decision aids are not readily available to individualize the benefits of smoking cessation but could help health care providers engage in meaningful conversations with their patients to explore and encourage an attempt to quit smoking. We conducted a pilot study of a novel decision aid among an underserved population to assess its effectiveness in increasing readiness to quit and quit attempts.

**Methods:**

We designed a decision aid that used images of birthday cakes to highlight the number of years of life that could be gained from smoking cessation and tested it in an urban safety net clinic. Active adult smokers were randomized to receive smoking cessation counseling, either with motivational interviewing techniques alone (control) or with motivational interviewing and the decision aid (intervention). The primary outcome assessed was readiness to quit, measured by using a previously validated contemplation ladder. The secondary outcome assessed was making a quit attempt.

**Results:**

Immediately following the interview, 21.1% of patients rose on the readiness-to-quit ladder; at 1 month, 40.6%; and at 3 months, 46.6%. We saw no significant difference between the control and intervention groups immediately after the interview (*P* = .79), at 1 month (*P* = .92), or at 3 months (*P* = .79). Over the 3-month follow-up period, 25% of patients in the control group made a quit attempt, and 15.4% of patients in the intervention group made a quit attempt (*P* = .30). Patients found the decision aid useful and easy to understand.

**Conclusion:**

Patients from an underserved population were highly receptive to a visual and personalized decision aid that highlighted the positive impact of smoking cessation. However, we found no difference in readiness to quit between patients who received motivational interviewing with the decision aid or without it.

## Introduction

From 1964 through 2012, the prevalence of cigarette smoking fell dramatically in the United States, from 42% to 18%, but progress has recently slowed (1). Forty-two million Americans continue to smoke, and large disparities in tobacco use exist by race/ethnicity, educational level, and socioeconomic status (2,3). The clinical encounter between a health care provider and patient can play an important role in promoting smoking cessation. Nonpharmacologic interventions, such as brief personalized advice, motivational interviewing, and use of office supports show varying levels of success in improving cessation rates (4–6).

Although not commonly used for smoking cessation, decision aids could help enhance the efficacy of nonpharmacologic interventions in the clinical encounter, particularly among patients who are less motivated (ie, precontemplative or contemplative) about changing their behavior. Decision aids have been shown to increase patient engagement, improve patient knowledge and perception of risk, reduce decisional conflict, and improve adherence to medication (7–10). Aids are an effective method of communicating health information to patients and can complement the principles of motivational interviewing. If decision aids are designed appropriately, they can also be effective tools among people with low health literacy or disadvantaged populations (11–13). 

To our knowledge, no available decision aids individualize the benefits of smoking cessation and focus on those benefits rather than focusing on the harms of continuing to smoke. Although considerable research describes the harms of smoking, including increased risk of disease and death related to cardiovascular disease, chronic obstructive lung disease, and lung cancer, emerging evidence has also begun to identify and quantify the benefits of cessation (14). Those who quit before the age of 35 or 40, for example, can avoid most of the excess mortality that comes from continuing to smoke (15–19).

We developed a novel decision aid and studied its use among smokers at an urban safety net clinic. The decision aid focused on the benefits of cessation rather than the harms of smoking and was designed to be both provocative and easy to understand. We hypothesized that a decision aid, when used in conjunction with motivational interviewing, could help health care providers engage in a meaningful conversation with their patients, advance patients’ readiness to quit, and ultimately encourage a quit attempt.

## Methods

We conducted a pilot study at an urban safety net clinic in Virginia. The clinic was located in an academic medical center with a predominantly underserved patient population. Patients were enrolled and completed follow-up over a course of 6 months, from October 1, 2016, through March 31, 2017. In this 2-arm controlled trial, patients in the control arm received motivational interviewing. Patients in the intervention arm received motivational interviewing and a decision aid that showed patients the benefits of smoking cessation (Figure). Interviews were conducted by 2 medical students who underwent formal training in motivational interviewing before patient enrollment.

**Figure:**
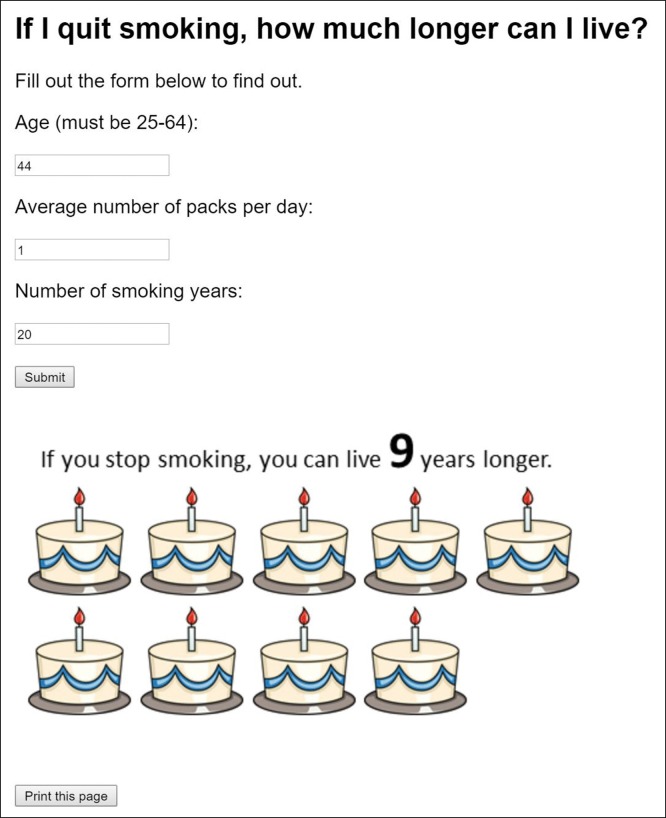
Screenshot of a web-based decision aid that highlights the benefits of smoking cessation. Medical students used the decision aid to engage with patients in a conversation about smoking cessation using motivational interviewing techniques.

Because it was meant to target a population with low health literacy, the decision aid was designed to be easy-to-understand, evocative, and memorable. It personalized and heightened the perceived benefits of quitting by using the poignant image of birthday cakes to display the potential years of life gained by quitting at the patient’s current age. To calculate the years of life gained by quitting, we used evidence from a study by Jha and colleagues, which showed that adults who quit smoking between the ages of 25 and 34 could gain 10 years of life, compared with those who continue smoking; similarly, adults who quit smoking between the ages of 35 and 44, 45 and 54, and 55 and 64 could gain 9 years, 6 years, and 4 years of life, respectively (19). The decision aid was readily accessible as a web application (http://smoking.skap.work/) and printable so that patients could take their personalized results home with them.

We targeted an enrollment of 100 active smokers with a 1:1 allocation ratio. One author (S.D.A.) used a random-number generator to place slips of paper labeled “intervention” or “control” into sequentially numbered, sealed envelopes for allocation concealment. Two medical students (M.K., J.M.) opened each envelope in sequential order as patients were enrolled, and patients were accordingly assigned to either the intervention or control arm on the basis of the slip of paper in the corresponding envelope. The study was not blinded.

### Patient population

We enrolled English-speaking men and women aged 25 to 64. Potential participants were identified by reviewing the clinic schedule for the day and subsequently approaching those who were listed in the electronic medical record as active or former tobacco users. Current smoking status was confirmed with the patient. Those who were active smokers were considered eligible for the study. Consent was obtained by using a verbal consent script.

### Measures

After randomization, all patients were given a 1-page questionnaire to obtain baseline characteristics, including smoking history (“At what age did you start smoking?” and “How many cigarettes do you smoke per day?”), and as assessment of their baseline readiness to quit. The questionnaire asked patients to place themselves on a readiness-to-quit ladder. The ladder scored from 1 to 10, where 1 indicated that the patient had no interest in quitting, 4 indicated that the patient sometimes thought about quitting but has no current plans to quit, and 8 indicated that the patient had begun to change, for example, by cutting back and setting a quit date. The contemplation ladder, which is based on stages of change (precontemplation, contemplation, preparation, action, and maintenance), is well validated (20–24). It captures meaningful changes in thinking that predict future quit attempts. After motivational interviewing, with or without use of the decision aid, patients were asked to place themselves again on the same readiness-to-quit ladder.

We gave an additional survey to patients in the intervention group to assess their receptiveness to the decision aid. On a 5-point Likert scale (strongly disagree, disagree, undecided, agree, and strongly agree) patients indicated how much they agreed with the following statements: “Before seeing the birthday cakes, I did not know that I could live longer if I quit smoking,” “That information was helpful and useful to learn,” and “The birthday cakes as a representation of how much longer I could live were easy to understand.”

One and 3 months later, we asked patients to again place themselves on a readiness-to-quit ladder. Patients were also asked whether they had made a successful quit attempt, even for a short time. For those patients in the intervention group who received the decision aid, we also asked, “Do you remember talking about how many extra years you can live if you quit smoking, displayed using birthday cakes?” The answer was recorded as yes or no.

### Statistical analysis

The primary outcome of our study was readiness to quit, assessed on a 10-point scale. The secondary outcome was making a quit attempt. We used χ^2^ analyses to compare proportions of patients in the intervention and control groups who rose on the readiness-to-quit ladder and the proportion of patients who made a quit attempt.

Analyses were conducted by using SAS, version 9.4 (SAS Institute, Inc.). All statistical tests were 2-tailed with a level of significance set at *P* < .05. The study was approved by the University of Virginia Institutional Review Board.

## Results

**Trial population and characteristics**. We randomized 100 patients to control and intervention groups. Five patients became unavailable for motivational interviewing between the time of randomization and their scheduled physician appointments. Final counts were 45 patients in the motivational interviewing-only group (control arm) and 50 patients in the motivational interviewing plus decision aid group (intervention arm). Baseline characteristics were similar between the 2 groups (Table 1). The average patient age was 49.7 years in the control group and 49.2 in the intervention group, and approximately 75% of patients in each group had either Medicaid or qualified for indigent care. The baseline score on the quit scale was 5.7 in the control group and 5.9 in the intervention group. A total of 34 patients in the control group (75.6%) and 35 patients in the intervention group (70.0%) completed follow-up at 1 month (Table 2). At 3 months, a total of 29 patients in the control group (64.4%) and 29 patients in the intervention group (58.0%) had completed follow-up.

**Table 1:** Characteristics of Patients (N = 95), Study of a Decision Aid to Encourage Smoking Cessation Among Patients at an Urban Safety Net Clinic, October 1, 2016–March 31, 2017^a^

Characteristic	Motivational Interviewing With Decision Aid (Intervention Group), n = 50	Motivational Interviewing Without Decision Aid (Control Group), n = 45
**Age, mean (SD), y**	49.2 (9.6)	49.7 (8.6)
**Sex**		
Male	20 (40.0)	27 (60.0)
Female	30 (60.0)	18 (40.0)
**Race**		
White	28 (56.0)	30 (66.7)
Nonwhite	22 (44.0)	15 (33.3)
**Insurance**		
Virginia indigent	26 (52.0)	22 (48.9)
Medicaid	12 (24.0)	11 (24.4)
Other	12 (24.0)	12 (26.7)
**Marital status**		
Married	9 (18.0)	1 (2.2)
Single or separated	22 (44.0)	25 (55.6)
Divorced or widowed	19 (38.0)	19 (42.2)
**Age when started smoking, mean (SD), y**	17.9 (6.6)	16.5 (4.8)
**Number of cigarettes smoked per day, mean (SD)**	13.4 (9.2)	16.4 (10.1)
**Baseline score on quit scale, mean (SD)**	5.9 (1.7)	5.7 (1.6)

Abbreviation: SD, standard deviation.

a Values are n (%) unless otherwise indicated.

**Table 2:** Readiness-to-Quit Status of Patients in Control (n = 45) and Intervention (n = 50) Groups, Study of a Decision Aid to Encourage Smoking Cessation Among Patients at an Urban Safety Net Clinic, October 1, 2016–March 31, 2017

Timing of Intervention	Increase in Readiness-to-Quit, %	No Change (or Decrease) in Readiness-to-Quit, %	*P* Value
**Immediately after interview**			
Motivational interviewing alone (control group) (n = 45)	22.2	77.8	.79
Motivational interviewing plus decision aid (intervention group) (n = 50)	20.0	80.0	
**1 month after interview**			
Motivational interviewing alone (control group) (n = 34)	41.2	58.8	.92
Motivational interviewing plus decision aid (intervention group) (n = 35)	40.0	60.0	
**3 months after interview**			
Motivational interviewing alone (control group) (n = 29)	44.8	55.2	.79
Motivational interviewing plus decision aid (intervention group) (n = 29)	48.3	51.7	

**Change in readiness to quit and quit attempts**. Immediately after the interview, 20 patients (21.1%) rose on the readiness-to-quit ladder; we found no significant difference between the control and intervention groups (*P* = .79) (Table 2). Among all patients with at least 1 month of follow-up, 40.6% rose on the readiness-to-quit ladder, 41.2% in the control group and 40.0% in the intervention group (*P* = .92). By 3 months, 46.6% of all patients rose on the ladder, 44.8% in the control group and 48.3% in the intervention group (*P* = .79). Over the 3-month follow-up period, 25% of patients in the control group and 15.4% of patients in the intervention group made a quit attempt (*P* = .30).

**Survey results**. Of patients who received the decision aid, 56% reported not knowing they could live longer if they quit smoking, 84% found the decision aid to be useful, and 100% found it easy to understand. Nearly all patients remembered the birthday cakes at follow-up.

## Discussion

In our study, we found that at 3 months, both motivational interviewing without the decision aid (control group) and with the decision aid (intervention group) improved patients’ readiness-to-quit and motivated quit attempts. However, we saw no significant difference in readiness to quit or quit attempts between the 2 groups. Decision aids have been shown to confer several benefits, such as improving patient knowledge and engagement. The lack of benefit seen in our study may be because both motivational interviewing and decision aids (used in a shared decision-making process) capitalize on the same mechanism: they both spur a meaningful conversation between health care providers and patients. Because our decision aid was designed to complement and build on the principles of motivational interviewing, it could, theoretically, be used independently to trigger a discussion between providers and their patients similar to that of motivational interviewing. For example, the practice of motivational interviewing involves developing a dissonance between a patient’s goals or values and his or her current behavior, adjusting to resistance rather than opposing it directly, and supporting self-efficacy and optimism (25). The decision aid attempts to encourage these 3 efforts by going beyond the harms of smoking and highlighting instead the benefits of quitting.

Because 75% of our study’s patients had either Medicaid or received free care because of indigent status, we targeted a truly vulnerable and underserved population. We demonstrated that such patients are highly receptive to a decision aid that is visual, personalized, and highlights the positive effect of smoking cessation. Months later, nearly all patients in the intervention group remembered talking about the potential years of life gained with smoking cessation, displayed as birthday cakes. We conducted motivational interviewing in a single session, but, because nearly all patients remembered the decision aid 3 months later, we might have seen greater gains by conducting additional motivational interviewing sessions and by using the decision aid as a launching point. The decision aid provided memorable teaching that health care providers could use to have a sustained dialogue with their patients about quitting.

Our study also showed that motivational interviewing can be effective even when done around the scheduled office visit and when conducted by someone other than a physician (eg, a medical student). Although providers frequently screen for smoking among their patients, they offer practical support for cessation less often (26,27). Involving other members of the clinical team to assist with motivational interviewing could increase the frequency of high-quality motivational interviewing in the setting of a busy clinical practice.

Our study had several limitations. First, as a pilot study, our sample size may have limited the power with which we could detect a significant difference between the 2 study groups. Second, the lack of blinding and the use of self-reporting of the outcomes could have biased our results. Some patients may have wanted to appear more willing and ready to quit. Third, we conducted only a single session of motivational interviewing and followed patients for only 3 months. Additional sessions and longer follow-up could have resulted in more dramatic changes and additional quit attempts. Fourth, as a single site study at an urban safety net clinic, our results may have limited generalizability. Finally, combining the decision aid with an already effective method (ie, motivational interviewing) may have diluted the effect of the decision aid. Future directions for this research include studying the effect of the decision aid alone or supplementing the effort with pharmaceutical interventions.

In conclusion, although we saw no difference in readiness to quit between patients who received motivational interviewing with a decision aid versus those who received motivational interviewing without a decision aid, patients from an underserved population were highly receptive to a visual and personalized decision aid that highlighted the positive impact of smoking cessation.
